# Pneumococcal polyarticular septic arthritis after a single infusion of infliximab in a rheumatoid arthritis patient: a case report

**DOI:** 10.1186/1752-1947-6-81

**Published:** 2012-03-09

**Authors:** Masatoshi Hayashi, Toshihisa Kojima, Koji Funahashi, Daizo Kato, Hiroyuki Matsubara, Tomone Shioura, Yasuhide Kanayama, Yuji Hirano, Masao Deguchi, Toshihisa Kanamono, Naoki Ishiguro

**Affiliations:** 1 Departments of Orthopedic Surgery and Rheumatology, Nagano Red Cross Hospital, 5-22-1 Wakasato, Nagano, 380-8582, Japan; 2 Departments of Orthopedic Surgery and Rheumatology, Nagoya University Graduate School of Medicine, 65 Tsuruma-cho, Showa-ku, Nagoya, 466-8550, Japan; 3 Department of Orthopedic Surgery, Shizuoka Kosei Hospital, 23 Kitaban-cho, Aoi-ku, Shizuoka, 420-8623, Japan; 4 Department of Orthopedic Surgery, Nagoya Kyoritsu Hospital, 1-172 Hokke, Nakagawa-ku, Nagoya, 454-0933, Japan; 5 Departments of Orthopedic Surgery and Rheumatology, Toyohashi Municipal Hospital, 50 Hachiken Nishi, Aotake-cho, Toyohashi, 441-8570, Japan

**Keywords:** infliximab, rheumatoid arthritis, septic arthritis, *Streptococcus pneumoniae*

## Abstract

**Introduction:**

We present a case of *Streptococcus pneumoniae *polyarticular septic arthritis in a patient with rheumatoid arthritis receiving a single infusion of infliximab.

**Case presentation:**

A 38-year-old Japanese man with a 5-year history of seronegative rheumatoid arthritis had previously received sulphasalazine and methotrexate therapies and was on regular low-dose prednisolone therapy. Despite these treatments, his disease activity remained high and infliximab was introduced in addition to methotrexate, prednisolone, and folic acid. However, he was admitted to hospital with a fever of 40.6°C, chills, and polyarthralgia eight days after the first infusion of infliximab. His joints were swollen, painful, and warm. Laboratory data showed marked acute inflammation. He was diagnosed with bacterial septic polyarthritis, and emergency surgical joint lavage and drainage was performed at the knees along with needle aspiration and lavage of the ankles and right wrist. He was then given intravenous antibiotic therapy for 31 days. He made a good recovery and was discharged on day 37.

**Conclusions:**

We believe this is the first reported case of severe pneumococcal septic arthritis requiring hospitalization in a patient treated with infliximab. *S. pneumonia *is now a well-recognized but uncommon cause of polyarticular septic arthritis that can lead to cessation of therapy, as in our patient's case.

## Introduction

Rheumatoid arthritis (RA) is a chronic, systemic inflammatory disease having a negative impact on the quality of life [1]. Anti-tumor necrosis factor (TNF) therapy is beneficial to RA patients because it suppresses inflammation and joint destruction [2]. Thus, the percentage of RA patients who are treated with anti-TNF agents is steadily increasing. Infliximab (IFX), an anti-TNF monoclonal antibody, exhibits excellent effectiveness in RA; however, many adverse events due to its use have been reported in patients. TNF is an important cytokine involved in initiating the protective immune response; therefore, patients receiving this therapy are at a high risk of infection. *Staphylococcus aureus *is the most common causative organism for septic polyarthritis with multiple joints. *Streptococcus pneumoniae *is rare (5% of septic arthritis cases) but is often responsible for polyarticular infections than other organisms [3]. We report a case of pneumococcal septic polyarthritis involving five joints in a patient with seronegative RA following a single infusion of IFX. This report suggests a possible association between the use of IFX and pneumococcal septic polyarthritis, a severe and often fatal infection.

## Case presentation

A 38-year-old Japanese man with seronegative RA, diagnosed in the year 2004 by using the American College of Rheumatology (ACR) 1987 criteria, had received sulphasalazine and methotrexate (MTX) therapies before he visited our institute and was started on regular low-dose prednisolone therapy. Despite these therapies, his RA disease activity remained high (tender and swollen joints at the knees and ankles; patient global assessment score, 48/100 mm; C-reactive protein (CRP), 3.37 mg/dL; erythrocyte sedimentation rate (ESR), 48 mm/hour; matrix metalloproteinase-3, 1531 ng/mL; Disease Activity Score 28-ESR, 4.57). The patient was obese (175 cm, 95 kg, BMI (body mass index): 31.0); however, he neither had a history of other medical problems (no viral infection and a non-carrier) nor had he ever received surgical intervention. He did not require intra-articular steroid injection. IFX at a dose of 3 mg/kg (total dose, 285 mg) was introduced in addition to MTX (10 mg weekly), prednisolone (5 mg daily), and folic acid (5 mg weekly). He suffered no immediate adverse effects and experienced excellent pain relief in his knees and ankles the day after his first infusion of IFX, but six days after infusion he presented with a fever of 40°C, chills, and polyarthralgia including the knees and ankles that persisted for two days, and he was admitted to hospital.

**Figure 1:**
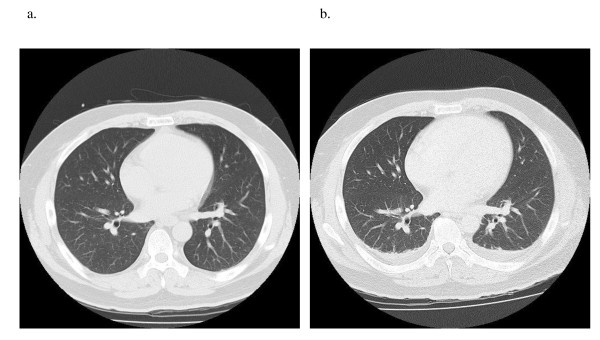
**(a) Computed tomography of the chest**. Hospital day 0. No abnormal findings. (**b**) Hospital day 2. Auxocardia and hydrothorax extending to both lungs.

**Figure 2:**
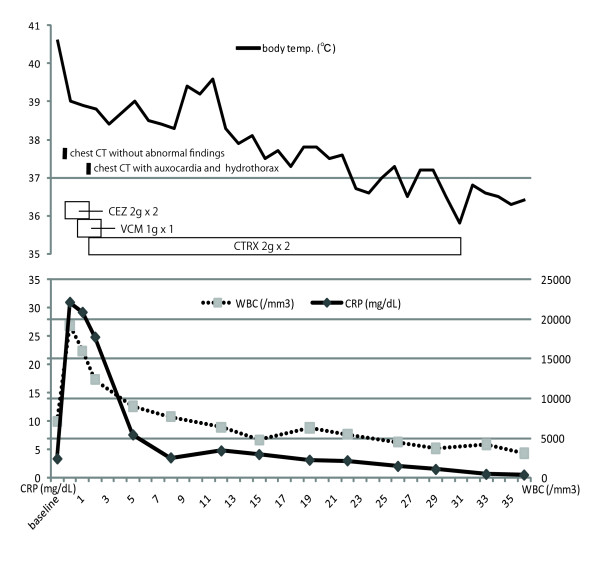
**Clinical course of the patient**. CT, computed tomography; CEZ, cefazolin; VCM, vancomycin; CTRX, ceftriaxone; WBC, white blood cells; CRP, C-reactive protein.

At admission (day 0, eight days after his first infusion), his body temperature was 40.6°C and he was tachycardic (107 beats/minute) with 104/68 mmHg blood pressure. His heart sounds were normal and he had no visible rash. The affected joints (knees, ankles, and right wrist) were swollen, painful, and warm. There was no evidence of a primary source of infection. Laboratory data showed marked acute inflammation (CRP, 31.0 mg/dL; white blood cells, 19,200/mm^3^) and pre-disseminated intravascular coagulopathy and shock (prothrombin time (PT), 62.2%; activated partial thromboplastin time (APTT), 45.1%; fibrinogen, 804 mg/dL; serum fibrin/fibrinogen degradation products (FDP), 14.7 μg/mL). Findings from chest computed tomography (CT) (Figure 1a), urine smears, and cultures were all normal, and he had no symptoms involving the abdomen or pelvis. Fluid drawn from the knees was purulent and its smear revealed numerous gram-positive cocci arranged in chains, which were subsequently identified as *S. pneumoniae *at day five. He was clinically diagnosed with bacterial septic polyarthritis. Emergency surgical joint lavage and drainage was then performed at the knees, along with needle aspiration and lavage of the ankles and right wrist just after admission (day 0). He was treated with intravenous antibiotic therapy with cefazolin (2 g twice daily at days 0 and one) and vancomycin (1 g once daily at days one and two). At day two, a whole body CT showed auxocardia and hydrothorax, but no pneumonia or other abnormal findings (Figure 1b). Two blood cultures taken at day 0 revealed penicillin-sensitive *S. pneumoniae *(minimal inhibitory concentration of penicillin G ≤ 0.03 μg/mL) with no penicillin-resistant strains at day two. He was switched to ceftriaxone (2 g twice daily at day two and thereafter for a total of 29 days). He made a rapid and sustained recovery with intensive rehabilitation. He was discharged on day 37 with 10 mg weekly of MTX, when he returned to his preadmission state (Figure 2).

## Discussion

IFX is a human-murine, chimeric, anti-TNF monoclonal antibody. It has been used in Japan since 2003 in the treatment of RA in conjunction with MTX. It is known to be very effective in decreasing inflammation, such as that in RA. However, it is also known that anti-TNF therapy may be a risk factor for a number of infections; in particular, IFX treatment is considered as a risk factor for reactivation of latent tuberculosis [4-6]. A post-marketing surveillance study in Japan showed that for RA patients, IFX in combination with low-dose MTX was well tolerated, but male gender, older age, Steinbrocker stage III or IV and comorbid respiratory disease were risk factors for bacterial pneumonia [7]. In addition, RA disease severity in itself is known to be one of the strong risk predictors of infection [8]. As mentioned above, patients treated with anti-TNF agents are generally believed to be at increased risk of bacterial infections [9]. Conversely, another study found that the severity of serious infections was not increased in anti-TNF-treated patients compared with a DMARD (disease modifying antirheumatic drugs)-treated cohort [10]. Considering the risks associated with the use of these agents, we were very careful when deciding whether to administer a particular biologic, and occasionally excluded immunocompromised patients who had comorbidities, such as diabetes mellitus, heart disease, viral hepatitis, and lung disease, or those who were of an advanced age, from these treatments. Although our patient was a 38-year-old man with RA, but otherwise healthy, and did not have any of the known risk factors for infection, he developed pneumococcal septic polyarthritis.

Upper and lower respiratory tract infection, ear and sinus infections, and meningitis are the most common manifestations of *S. pneumoniae *infection [11]. Recently, the emergence of *S. pneumoniae *strains with reduced susceptibility to penicillin has been increasing. These strains account for 2% t0 3% of cases [12-15]. Third-generation cephalosporins are the preferred first-line treatment until reduced susceptibility to penicillin is ruled out [11], but given the severity (possible sepsis or concurrence of meningitis) and the less frequent target (joints) of our patient's infection, we did not rule out the presence of other strains with reduced susceptibility to penicillin such as PISP or PRSP (penicillin-intermediate or penicillin-resistant *S. Pneumonia*). As a result we first selected intravenous antibiotic therapy with the third-generation cephalosporin ceftriaxone, rather than penicillin G [11]. Therefore, from day two onwards, we used ceftriaxone, which was effective as mentioned above. We used vancomycin with ceftriaxone to control septic arthritis until sensitivity to ceftriaxone was confirmed on day two, but after that we stopped the use of vancomycin.

*S. aureus *is the most common causative organism in septic arthritis in RA [16], and polyarticular septic arthritis in RA patients has a 50% mortality rate [3]. The bones and joints are less frequently infected by *S. pneumoniae*, which causes 3% to 10% of the septic arthritis cases [12-14,17-19]. *S. pneumoniae *is more often responsible for polyarticular infections than other organisms [3]. Preceding or concurrent foci of extra-articular infection are common in patients with pneumococcal septic arthritis, probably because of other bacterial causes of joint infection. Bacteremia and pneumonia are the most common extra-articular sites of pneumococcal infection [13]. However, our patient had no previous or coexisting infection such as pneumonia (Figure 1a, b). Because a recent cohort study showed that the adjusted hazard ratio for septic arthritis in patients treated with anti-TNF agents was 2.3 (95% confidence interval (CI) 1.2 to 4.4) and hazard estimates for the first year of follow-up were found to be increased in anti-TNF treatment [16], it is especially important to carefully monitor early RA patients given anti-TNF agents.

## Conclusions

To the best of our knowledge, this is the first reported case of pneumococcal septic polyarthritis following treatment with IFX in a patient with RA. To avoid aggravation of a patient's illness and to prevent death due to severe infection, it is important for clinicians to be aware of the fact that use of TNF-inhibitors can lead to infections in locations such as joints. Infections at these locations are difficult to distinguish from the symptoms of a flare-up of RA. Because delay in controlling an infection worsens a patient's RA disease activity during that period, it is important to get any infection rapidly under control. However, it is impossible to avoid accidental infections such as in this case, irrespective of the level of precautions. Recently, rheumatic treatment is being rapidly adopted based on the concept of 'treat-to-target' and therefore, we need to accumulate evidence from case studies to promote safe and effective treatments for RA and to avoid diagnostic delays and therapeutic errors to achieve maximum possible survival of patients.

## Consent

Written informed consent was obtained from the patient for publication of this case report and any accompanying images. A copy of the written consent is available for review by the Editor-in-Chief of this journal.

## Competing interests

None of authors are industry employees. TK and NI have received lecture fees from Abbott Japan Co., Ltd.; Bristol-Meyers Squibb Co.; Chugai Pharmaceutical Co., Ltd.; Eisai Co., Ltd.; Mitsubishi Tanabe Pharma Corporation; Pfizer Japan Inc.; and Takeda Pharmaceutical Co., Ltd.

## Authors' contributions

MH analyzed and interpreted the patient data, and was a major contributor in writing the manuscript. All authors read and approved the final manuscript.
